# A 3D Poly(ethylene glycol)-based Tumor Angiogenesis Model to Study the Influence of Vascular Cells on Lung Tumor Cell Behavior

**DOI:** 10.1038/srep32726

**Published:** 2016-09-06

**Authors:** Laila C. Roudsari, Sydney E. Jeffs, Amber S. Witt, Bartley J. Gill, Jennifer L. West

**Affiliations:** 1 Department of Biomedical Engineering, Duke University, Durham, North Carolina, USA; 2 Department of Bioengineering, Rice University, Houston, Texas, USA

## Abstract

Tumor angiogenesis is critical to tumor growth and metastasis, yet much is unknown about the role vascular cells play in the tumor microenvironment. *In vitro* models that mimic *in vivo* tumor neovascularization facilitate exploration of this role. Here we investigated lung adenocarcinoma cancer cells (344SQ) and endothelial and pericyte vascular cells encapsulated in cell-adhesive, proteolytically-degradable poly(ethylene) glycol-based hydrogels. 344SQ in hydrogels formed spheroids and secreted proangiogenic growth factors that significantly increased with exposure to transforming growth factor beta 1 (TGF-β1), a potent tumor progression-promoting factor. Vascular cells in hydrogels formed tubule networks with localized activated TGF-β1. To study cancer cell-vascular cell interactions, we engineered a 2-layer hydrogel with 344SQ and vascular cell layers. Large, invasive 344SQ clusters (area > 5,000 μm^2^, circularity < 0.25) developed at the interface between the layers, and were not evident further from the interface or in control hydrogels without vascular cells. A modified model with spatially restricted 344SQ and vascular cell layers confirmed that observed cluster morphological changes required close proximity to vascular cells. Additionally, TGF-β1 inhibition blocked endothelial cell-driven 344SQ migration. Our findings suggest vascular cells contribute to tumor progression and establish this culture system as a platform for studying tumor vascularization.

Tumor angiogenesis is the process of blood vessel recruitment to a tumor site in order to support delivery of nutrients and removal of waste^1^,^2^. This blood vessel supply enables rapid tumor growth and facilitates metastasis, the leading cause of death from cancer, via entry of cancer cells into the bloodstream^3^. Because of its significance in the tumor progression process, a complete understanding of tumor neovascularization and the influence of vascular cells on tumor cell behavior is essential to the development of therapeutics that effectively target the tumor vasculature.

A major outstanding challenge associated with studying tumor angiogenesis is that existing preclinical models are limited in their recapitulation of *in vivo* cellular organization in 3D. This highlights the need for better approaches to study the dynamic interplay of relevant cells and signaling molecules as they are organized in the tumor microenvironment^2^,^4^. Tumor angiogenesis has traditionally been studied with the use of *in vivo* mouse models and 2D *in vitro* culture systems^2^. The major advantage of using *in vivo* models is that all physiological systems are present and functional, providing a complete representation of tumor heterogeneity and relevant interactions that occur on the molecular, cellular, and organ system level. However, this complexity makes it difficult to elucidate or manipulate the roles of specific tumor components, such as isolation of the role of tumor cell-endothelial cells interactions in tumor progression. *In vitro* models often enable researchers to isolate individual parameters of interest, but relevant interactions are sometimes lost^2^. Additionally, while most *in vitro* culture systems examine cells grown on 2D surfaces, research has shown that cells often respond differently in 2D versus 3D culture^5^,^6^,^7^,^8^. When studying angiogenesis, the need for 3D culture systems is even more pronounced as endothelial tubulogenesis is required.

Tissue engineering approaches have been applied to the development of 3D tumor organ models where cancer cells are incorporated into scaffold materials such as Matrigel^9^,^10^,^11^, collagen^12^, or a combination of both^13^. In considering choices of scaffold materials for *in vitro* tumor models, one seeks control over cell adhesion and signaling, a platform that supports cell migration in 3D, and tunable scaffold mechanical properties. One scaffold material that meets these criteria is poly(ethylene glycol) (PEG), used in tissue engineering for its biocompatibility, ease of crosslinking to create a hydrogel, and finely tunable bioactivity^14^. Due to the hydrophilic, neutral properties of PEG and its high chain mobility, it inherently resists protein adsorption and subsequent cell adhesion^15^,^16^. Peptides and growth factors can be covalently immobilized within PEG hydrogels to customize the cellular microenvironment that is otherwise absent of bioactivity^17^,^18^,^19^,^20^. Additionally, PEG can be rapidly crosslinked via photopolymerization, which allows for 3D encapsulation of cells with high viability^17^.

Cells derived from a murine KRas- and p53-mutant lung adenocarcinoma model, known as 344SQ^9^,^21^, have previously been incorporated in 3D in Matrigel^9^ and PEG-based hydrogels^14^ to explore the influence of extracellular matrix (ECM) on tumor progression and metastasis. While 344SQ are widely metastatic *in vivo*, when cultured in 3D *in vitro* they revert to an epithelial phenotype^9^,^14^. 344SQ form lumenized spheres with epithelial polarity that mimic the structure of normal lung acini, and can be perturbed to transition to a metastatic, mesenchymal phenotype by exposure to transforming growth factor beta 1 (TGF-β1)^9^,^14^. This behavior is characteristic of the epithelial-to-mesenchymal transition (EMT), a process that occurs in normal tissue development. EMT is harnessed by non-migratory epithelial cell-derived tumor cells *in vivo* to facilitate dissemination from the primary tumor site, making it a target for development of novel therapeutics^14^,^22^,^23^. This phenotypic plasticity makes 344SQ an attractive cancer cell source to study tumor progression-promoting factors in the context of tumor angiogenesis.

Previous work has also shown that endothelial cells and pericytes co-cultured in cell-adhesive and proteolytically-degradable PEG-based hydrogels form robust microvascular networks that are stable in culture for at least 28 days^17^. Cells within these networks form lumenized tubes and secrete ECM components to build the basal lamina that typically surrounds microvascular networks^17^. Additionally, this microvascular system has been incorporated into a microfluidic device, and successful perfusion of the networks within the device has been demonstrated, validating their functionality^24^. The current report seeks to combine 3D culture of 344SQ with adjacent 3D microvascular cultures in PEG-based hydrogels to study tumor angiogenesis and the impacts of neovascularization on tumor cell behavior. The tumor angiogenesis model developed in this work supports the study of many relevant aspects of tumor vascularization in 3D: angiogenic paracrine signaling from cancer cells, the EMT-promoting potential of vascular cells, and the impact of soluble versus direct vascular cell-cancer cell interactions on 3D collective cancer cell invasion.

## Results

### Material Synthesis and Characterization

Fabrication of cell-adhesive, proteolytically-degradable PEG hydrogels first required the conjugation of bioactive peptides to monoacrylate PEG-succinimidyl valerate (PEG-SVA) via nucleophilic substitution reactions (Supplementary Fig. S1). The peptide RGDS, a cell-adhesive peptide derived from fibronectin, was conjugated to PEG to yield PEG-RGDS. GGGPQGIWGQGK (abbreviated as PQ), a MMP-2 and -9 sensitive degradation sequence found in the alpha chain of type I collagen, was also reacted with PEG-SVA and PEG chains flanked both ends of the peptide to yield PEG-PQ-PEG^25^. Synthesis of PQ was confirmed with MALDI-ToF mass spectrometry (Supplementary Fig. S2) and conjugation of the RGDS and PQ peptides to PEG-SVA was confirmed using gel permeation chromatography. Successful conjugation was demonstrated by the increased molecular weight of PEG-peptide samples compared to unconjugated PEG-SVA samples (Supplementary Fig. S2). Typical elution times for PEG-PQ-PEG, PEG-RGDS, and PEG-SVA were 6 min, 6.4 min, and 6.9 min, respectively. The conjugation efficiency was greater than 85% for all samples.

### Evaluation of Cancer and Vascular Hydrogels Independently

**Figure 1:**
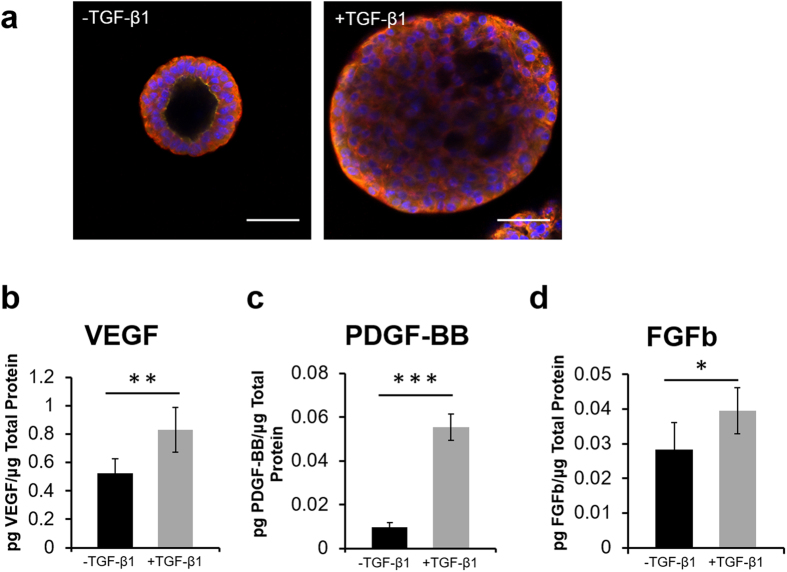
Characterization of the behavior of cells in C-hydrogels and assessment of growth factor secretion profiles from C-hydrogels. (**a**) 344SQ sphere formation (left) and response to TGF-β1 treatment (right) (red – β-catenin, yellow – ZO-1, blue – DAPI; scale bars = 50 μm). (**b**) Angiogenic growth factor secretion from 344SQ with and without TGF-β1 treatment (black – no TGF-β1, gray – 5 ng/mL TGF-β1; n = 6 hydrogels; values are reported as mean ± s.d.; *indicates statistical significance as determined by a two-tailed t-test: *p < 0.05, **p < 0.01, ***p < 0.001).

**Figure 2:**
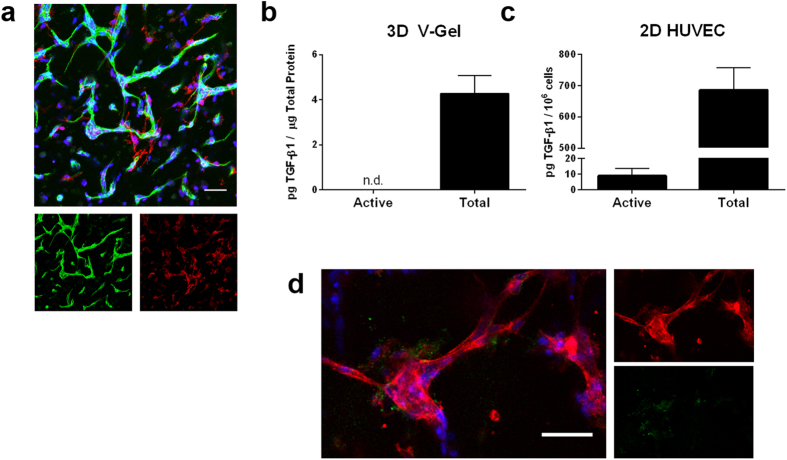
Characterization of the behavior of cells in V- hydrogels and assessment of TGF-β1 growth factor secretion from vascular cells. (**a**) Network formation in vascular hydrogels (green – PECAM; red – α-smooth muscle actin; blue – DAPI). (**b**) Active and latent TGF-β1 secretion from V hydrogel (n.d. = not detected; n = 5 hydrogels; values reported as mean ± s.d.). (**c**) Active and latent TGF-β1 secretion from 2D seeded HUVEC (n = 3 wells (active) and n = 4 wells (total); values reported as mean ± s.d.). (**d**) Immunostaining of active TGF-β1 shown to be localized around vascular cell networks in PEG hydrogels (red – phalloidin, blue – DAPI, green – active TGF-β1; scale bars = 50 μm).

344SQ lung adenocarcinoma cells were encapsulated in bioactive PEG-based hydrogels (referred to as C-hydrogels). Images confirmed formation of lumenized spherical cell clusters with epithelial morphology as indicated by staining with epithelial polarity markers, β-catenin and ZO-1, as previously described (Fig. 1a, Supplementary Fig. S3)^14^. Following treatment with TGF-β1, both lumenization and epithelial polarity were lost, and clusters increased in size. A co-culture of human umbilical vein endothelial cells (HUVEC) and human vascular pericytes (HVP) were encapsulated in bioactive PEG-based hydrogels (referred to as V-hydrogels). Images confirmed the formation of networks (Fig. 2a).

### Growth Factor Secretion

Angiogenic growth factor secretion from C-hydrogels was assessed via ELISA (Fig. 1b–d). VEGF, PDGF-BB, and bFGF were present in the conditioned media (523.6 ± 103.7, 9.6 ± 2.1, and 26.3 ± 9.3 pg protein/mg total protein, respectively) and following treatment with TGF-β1, 344SQ secreted significantly higher concentrations of all 3 growth factors assessed (830.2 ± 158.5, 55.5 ± 6.0, and 39.6 ± 6.7 pg protein/mg total protein; *p* = 0.004, *p* < 0.0001, and *p* = 0.02, respectively).

In order to assess the EMT-inducing capability of the vessel cell co-culture, active and total TGF-β1 ELISAs were performed on the conditioned media from V-hydrogels (Fig. 2b). Results showed the vascular cells secreted 1.5 ± 0.16 ng/mL total TGF-β1 (4,270 ± 810 pg TGF-β1/ mg total protein) and undetectable levels of active TGF-β1. Due to the undetectable levels of active TGF-β1 in conditioned media from 3D V-hydrogels, an ELISA was also performed on 2D seeded HUVEC alone and revealed 8.8 ± 4.5 pg active TGF-β1/10^6^ cells and 685.9 ± 71.3 pg total TGF-β1/10^6^ cells in the conditioned media (Fig. 2c), indicating endothelial cells are capable of producing activated TGF-β1 outside of a hydrogel system. Overall this data suggests that relevant angiogenic and EMT-inducing growth factors were secreted independently by the cancer cells and vascular cells in 3D.

### Active TGF-β1 Immunostaining

Due to the observed differences in 2D and 3D TGF-β1 secretion from vascular cells, it was hypothesized that the lack of active TGF-β1 detected in V-hydrogel conditioned media was in part due to sequestration of TGF-β1 in ECM proteins secreted by vascular cells. It is known that secreted TGF-β1 is often sequestered in the ECM and cells in V-hydrogels secrete ECM around microvascular networks^17^,^26^. Immunostaining of cells within tumor angiogenesis models using an antibody specific for active TGF-β1 revealed the presence of positive active TGF-β1 staining localized around vascular cell networks (Fig. 2d).

### Characterization of Cancer and Vascular Cell Migration Behavior

**Figure 3:**
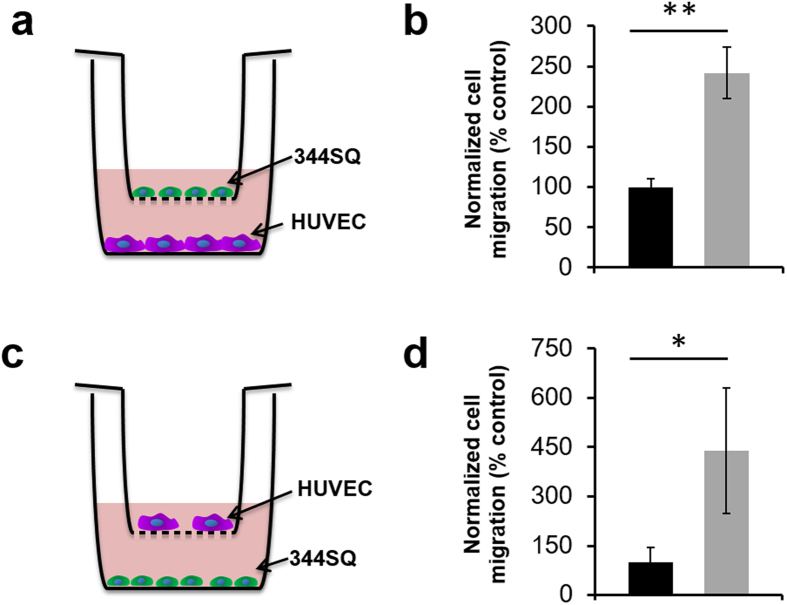
Migration behavior of 344SQ and HUVEC. (**a**) Schematic of study setup for 344SQ migration. (**b**) 344SQ migration was significantly enhanced in the presence of HUVEC over the media only control (black – media only in bottom well, gray – seeded cells in bottom well; 5 images taken per insert as repeated measures, n = 12 inserts per group; values reported as mean ± s.d.). (**c**) Schematic of study setup for HUVEC migration. (**d**) HUVEC migration was significantly enhanced in the presence of 344SQ over the media only control (black – media only in bottom well, gray – seeded cells in bottom well; 5 images were taken per insert as repeated measures, n = 6 inserts per group; values reported as mean ± s.d.). (*indicates statistical significance as determined by a two-tailed t-test, in all cases p < 0.01).

To evaluate the potential stimulatory effects of vascular cell interactions on 344SQ, migration in response to soluble signals from HUVEC was assessed using a transwell assay (Fig. 3a). After 16 hr of incubation on the inserts, 344SQ exhibited a 140% increase in migration over control (*p* = 0.001, Fig. 3b). HUVEC migration in response to soluble signals from 344SQ was also assessed (Fig. 3c). Following 16 hr of incubation with 344SQ, HUVEC migration on transwell inserts was shown to have increased by greater than 300% compared to the control (*p* = 0.007, Fig. 3d).

### Tumor Angiogenesis Model Design and Validation

**Figure 4:**
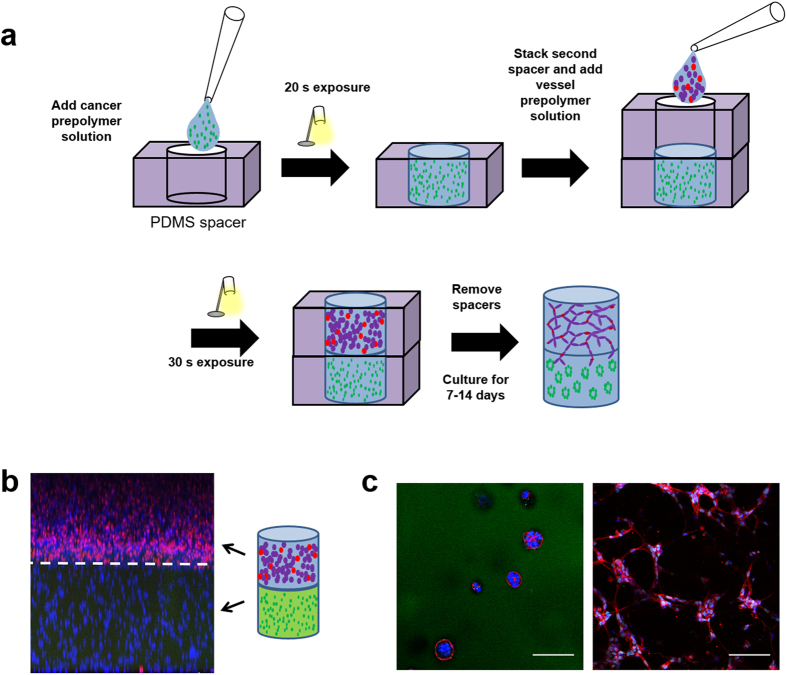
Tumor angiogenesis model construction schematic and model validation. (**a**) Cancer prepolymer solution is added to a PDMS spacer well, followed by a 20 s photopolymerization. A second PDMS spacer well is stacked on top of the initial spacer well, followed by a 30 s photopolymerization and removal of PDMS spacer wells. (**b**) Image of V-C hydrogel on day 0 in culture showing vascular cells and cancer cells in their respective regions of the hydrogel (red – human nuclei, blue – DAPI, green – Alexafluor 488-PEG-RGDS). (**c**) Images of V-C hydrogel at day 3 in culture showing cancer cells forming spherical clusters in the fluorescently tagged region of the hydrogel (left, 2 μm-thick slice) and vascular cells forming tubule networks in the non-fluorescently tagged region of the hydrogel (right, 30 μm-thick z projection) (red – phalloidin, blue – DAPI, green – Alexafluor 488-PEG-RGDS, cyan – human nuclei and PECAM; scale bars = 100 μm).

Fabrication of the tumor angiogenesis model is depicted in Fig. 4a, whereby sequential addition of cancer and vascular cell-laden prepolymer solutions into stacked PDMS wells enabled the formation of a bilayer hydrogel. Bilayer hydrogels comprised of vascular cell and cancer cell layers are abbreviated as V-C hydrogels and control bilayer hydrogels comprised of an acellular “blank” hydrogel layer and a cancer cell layer are abbreviated as B-C hydrogels. Previous work has reported that 344SQ can be polymerized in PEG hydrogels for 30 s and retain high viability^14^. To ensure the double polymerization (50 s total white light exposure) did not negatively affect 344SQ viability, Live/Dead staining of 344SQ in B-C hydrogels was performed after 48 hr in culture. Imaging revealed high 344SQ viability was retained (Supplementary Methods and Supplementary Fig. S4). HUVEC have also been previously encapsulated in PEG hydrogels with a 30 s polymerization time so we did not perform viability testing for our vascular cell co-culture as the polymerization time was the same for V-C hydrogels (30 s)^18^. Fabrication of the tumor angiogenesis model was validated with the use of immunocytochemistry and confocal microscopy (Fig. 4b,c). Fluorescent PEG-RGDS was added to the cancer cell-laden prepolymer solution to enable visualization of the interface, and a human nuclear antibody was used to identify the vascular cells. For hydrogels fixed on day 0 in culture, fluorescent PEG-RGDS was localized in the cancer hydrogel and human nuclear staining was localized in the vascular hydrogel, indicating successful fabrication of a 2-layer hydrogel with distinct cell compositions in each layer (Fig. 4b). V-C hydrogels were also fixed on day 3 and imaging revealed the formation of small cancer clusters in the cancer hydrogel layer and vascular network structures forming in the vascular hydrogel layer (Fig. 4c, Supplementary Fig. S4).

### Evaluation of Cancer Cell Morphology Changes in Tumor Angiogenesis Model

**Figure 5:**
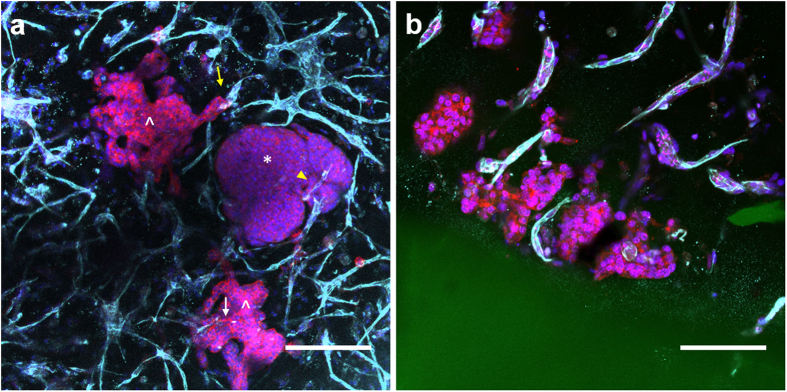
Images of cancer cells and vascular cells in V-C hydrogels interacting at the hydrogel interface at day 12. (**a**) A view of the hydrogel parallel to the interface (*indicates large, disorganized clusters, ^ indicates invasive clusters, yellow ^↑^indicates vascular cells in contact with the tip of invasive projects, white ^↑^indicates vascular cell invasion into a cancer cluster, yellow ▴ indicates clefts in clusters; red – phalloidin, blue – DAPI, cyan – PECAM and human nuclear antibody, green – Alexafluor 488-PEG-RGDS; scale bar = 200 μm). (**b**) A view of the hydrogel orthogonal to the interface (red – phalloidin, blue – DAPI, cyan – PECAM and human nuclear antibody, green – Alexafluor 488-PEG-RGDS; scale bar = 100 μm).

Over time in V-C hydrogels, it was observed that some 344SQ clusters at the hydrogel interface were large and disorganized (indicated by *, Fig. 5a) and in several cases exhibited invasive projections in the surrounding hydrogel (indicated by ^, Fig. 5a). This morphology stood out from that previously observed for 344SQ in PEG hydrogels: small, spherical structures with epithelial morphology and cleared lumens^14^. The term “cluster” was chosen to refer to aggregates of 344SQ cells formed in PEG hydrogels, whether they were spherical or invasive in morphology, and this terminology will be used henceforth. Vascular cells in V-C hydrogels, as identified by PECAM and human nuclear antibody staining, were observed to home around 344SQ clusters at the interface and interact with them (Fig. 5a,b). Vessel cell-cancer cell interactions were primarily localized around the periphery of invasive 344SQ cell clusters and a few examples of vascular cells penetrating through clusters were observed (indicated by white arrow, Fig. 5a). In many cases, vascular structures were observed to be in contact with the tip of invasive cancer projections (indicated by yellow arrow, Fig. 5a) or located in clefts in large, disorganized structures (indicated by yellow arrowhead, Fig. 5a). While it is apparent the latter occurred as a result of vascular invasion, live imaging would be necessary to confirm how the vascular cell-cancer cell interactions at the tips of invasive projections are initiated. However, based on our migration data, we hypothesize these interactions transpire as a result of both vascular cell and cancer cell migration. Additionally, vascular structures in V-C hydrogels were confirmed to have lumen as our lab has previously reported to be typical for vascular cells in PEG hydrogels (Supplementary Fig. S5)^24^.

**Figure 6:**
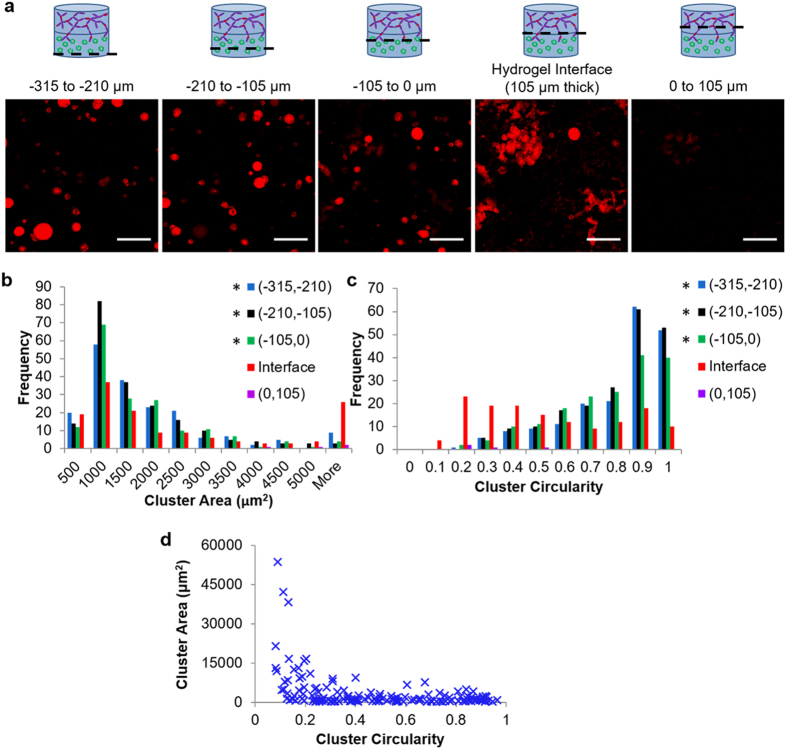
Analysis of 344SQ morphology changes based on location in V-C hydrogels. (**a**) Schematic and images of 105-μm thick bins of the cancer region of the hydrogel (red – phalloidin; scale bars = 200 μm). (**b**) Cluster area histogram broken down into bins. (**c**) Cluster circularity histogram broken down into bins. For (**b**,**c**), *indicates statistical significance as compared to the interface group as determined by Kolmorogov-Smirnov tests: p < 0.0125 (Bonferroni corrected); 3 images were taken per hydrogel as repeated measures across 5 hydrogels, 739 total clusters analyzed. (**d**) Cluster area versus circularity for all clusters imaged in the hydrogel interface bin.

To confirm the regional occurrence of large, invasive structures in V-C hydrogels, z-stacks obtained were used to divide the hydrogel into bins that were 105-microns thick (Fig. 6a, Supplementary Fig. S6). Quantification of cluster area was performed for each of the bins of vascular and cancer hydrogels (Fig. 6b). This analysis revealed that the population of large clusters with projected area greater than 5,000 μm^2^ in the interface bin of the tumor angiogenesis model was higher than in the other regions of the hydrogel. Quantification of cluster circularity was also performed for each bin, and this analysis revealed an enhanced frequency of clusters with low circularity (<0.25) for the interface region that was not observed in the other hydrogel bins (Fig. 6c). Furthermore, the distribution of clusters in the interface bin was significantly different than the 3 bins below it for both area and circularity (area: *p* = 0.002 compared to (−105, 0), *p* = 0.002 compared to (−210, −105), and *p* = 0.007 compared to (−315, −210); circularity: *p* < 0.0001 for all 3 regions). Area and circularity plotted for each cluster at the interface confirmed the clusters with large area also exhibited low circularity (Fig. 6d). Taken together, area and circularity analysis supports the hypothesis that large, invasive structures only occurred at the hydrogel interface where interactions were occurring between cancer cells and vascular cells.

**Figure 7:**
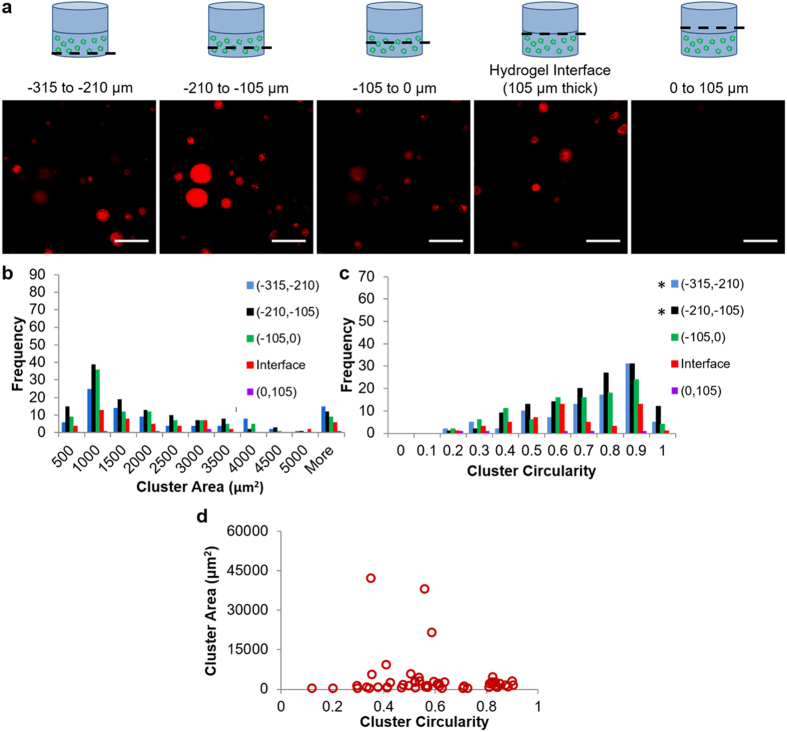
Analysis of 344SQ morphology changes based on location in B-C hydrogels. (**a**) Schematic and images of 105-μm thick bins of the cancer region of the hydrogel (red – phalloidin; scale bars = 200 μm). (**b**) Cluster area histogram broken down into bins. (**c**) Cluster circularity histogram broken down into bins. For (**b**,**c**), *indicates statistical significance as compared to the interface group as determined by Kolmorogov-Smirnov tests: p < 0.0125 (Bonferroni corrected); 3 images were taken per hydrogel as repeated measures across 5 hydrogels, 415 total clusters analyzed. (**d**) Cluster area versus circularity for all clusters imaged in the hydrogel interface bin.

As a control, cluster area and circularity were also evaluated for B-C hydrogels (Fig. 7). This analysis revealed a lack of structures with high area or low circularity occurring at the hydrogel interface (Fig. 7b,c). For cluster area, no significant differences in cluster distribution in the interface were observed compared to the other bins. For cluster circularity, the (−315, −210) and (−210, −105) bins were significantly different from the interface bin (*p* = 0.0005 and *p* = 0.0006, respectively). However, in contrast to the interface bin for the V-C hydrogels where there was a cluster population shift to clusters with lower circularity, the interface bin for the B-C hydrogels did not exhibit a shift to clusters with lower circularities. Rather, the significant difference is likely attributed to the decrease in clusters with 0.7–0.8 circularity. In addition, area and circularity plotted for each cluster at the interface confirmed the clusters with large area did not also exhibit low circularity (Fig. 7d).

### 3D Transwell Signaling Study

**Figure 8:**
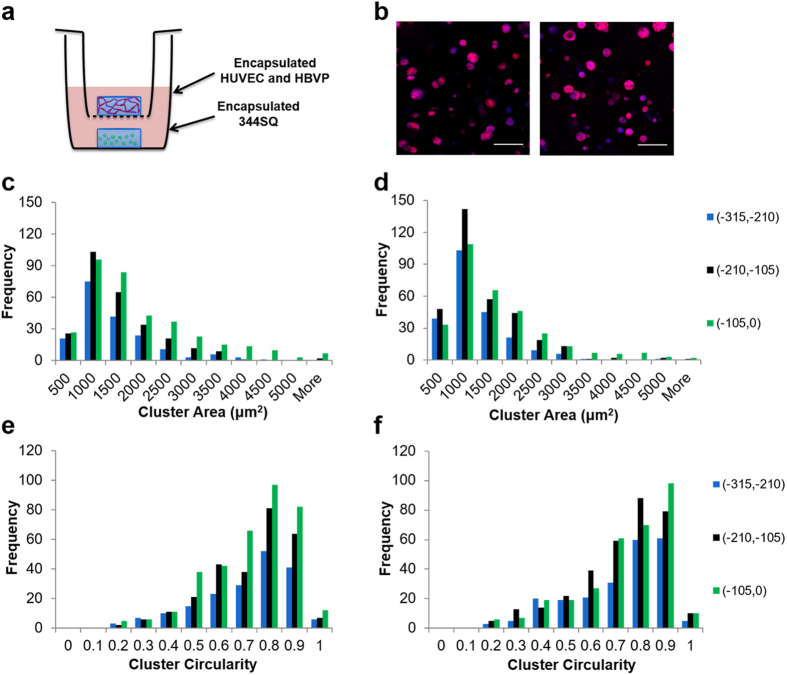
Analysis of 344SQ behavior restricted to soluble signaling from vascular cells in a 3D transwell assay. (**a**) Schematic of the study setup. (**b**) Representative images of spheres in (0, −105) μm region of the hydrogels (left – no vessel, right – with vessel; red – phalloidin, blue – DAPI; scale bars = 200 μm). Histogram of cluster area broken down into bins according to distance from the top of the hydrogel for (**c**) no vessel group and (**d**) with vessel group. Histogram of cluster circularity broken down into bins according to distance from the top of the hydrogel for (**e**) no vessel group and (**f**) with vessel group. No significant differences compared to the corresponding no vessel group as determined by Kolmorogov-Smirnov tests at p < 0.0167 (Bonferroni corrected); 4 images were taken per hydrogel as repeated measures, n = 4 hydrogels per group.

Due to the findings that large, invasive clusters occurred in the interface region of the hydrogels and required the presence of vascular cells, it was hypothesized that changes in cancer cluster morphology required close proximity to vascular cells. A modified tumor angiogenesis model was developed to test this hypothesis. A cancer hydrogel was cultured in the same well as a vascular hydrogel placed in a transwell insert (Fig. 8a). This model setup facilitated soluble signaling between cells in the 2 hydrogels but did not allow for direct cellular interaction between cells in the separate hydrogels. Analysis of cluster area and cluster circularity throughout the hydrogel thickness revealed no significant differences in the distribution of clusters comparing the treatment to the no vessel corresponding bin for either metric assessed (Fig. 8b–f), indicating the necessity for vascular cells to interact closely with cancer cells to drive changes in cancer cell behavior.

### TGF-β1 Inhibitor Migration Assay

**Figure 9:**
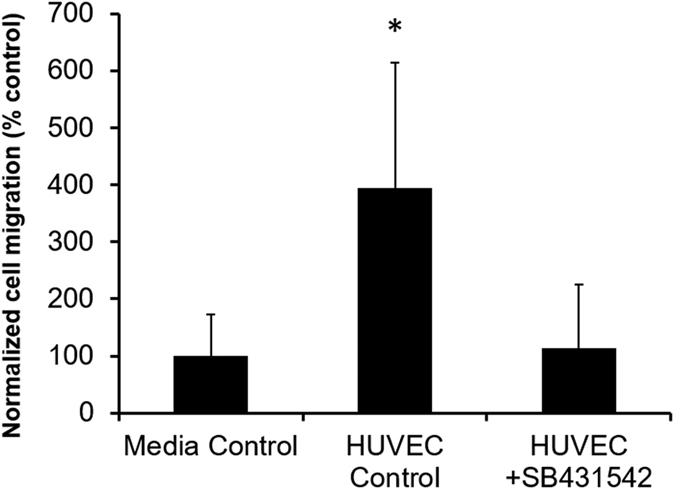
Vascular cell-secreted active TGF-β1 as a mechanism for 344SQ invasive behavior. 344SQ migration in the presence of seeded HUVEC and SB541542, a TGF-β1 inhibitor, was significantly decreased compared to the seeded HUVEC positive control (5 images were taken per insert as repeated measures, n = 5 inserts per group; values are reported as mean ± s.d.; *indicates statistical significance determined by a one-way ANOVA and subsequent Tukey-Kramer HSD tests, p < 0.05 versus all others).

A 344SQ migration assay in the presence of HUVEC using treatment with a TGF-β1 inhibitor, SB431542, was performed to investigate the hypothesis that vascular cell secreted TGF-β1 was contributing to changes in 344SQ behavior. SB431542 is a small molecule that inhibits TGF-β superfamily type 1 activin receptor-like kinases-4,-5, and -7^27^. Inhibition of these receptors prevents TGF-β-induced phosphorylation of Smad2 and Smad3, which can lead to EMT occurrence downstream^27^,^28^. Treatment with SB431542 significantly decreased the number of migrated 344SQ by greater than 280% as compared to the HUVEC control group (Fig. 9, *p* = 0.03). Additionally, 344SQ migration in the presence of SB431542 was not significantly different from migration in the media only control group, indicating TGF-β1 inhibition alone was able to decrease migration down to a level comparable to that of no HUVEC.

## Discussion

The goal of this work was to design and develop a tumor angiogenesis model comprised of 2 PEG-based hydrogel layers, each with different cell compositions: a cancer cell monoculture (344SQ) and a vascular cell co-culture (HUVEC and HVP). The resulting tumor angiogenesis model we developed is the first synthetic material platform used to spatially localize cancer and vascular cells. The only other reported synthetic tumor angiogenesis model we found was a tri-culture of homogeneously mixed cancer cells, endothelial cells, and mesenchymal stem cells^29^. We aimed to improve upon previously reported approaches in part by defining cellular localization within our system to better match that which occurs physiologically. Our dual layer approach allowed vascular cells to undergo tubulogenesis and cancer cells to form tumor spheroids in separate layers. Secreted growth factors diffused freely through the hydrogels and engineered cell-mediated degradability of the PEG matrix allowed both vascular cells and cancer cells to migrate and interact with each other. Combined, these features expanded the capabilities of current synthetic systems by supporting tumor-driven vascular cell homing to cancer cell aggregates and study of cell-cell interactions that are mediated by the cells themselves.

Other models that have spatially restricted cancer cells and vascular cells at study initiation have made use of natural materials, where cancer cells were cultured in 3D with endothelial cells seeded in 2D on top in collagen^30^,^31^ or Matrigel^32^ and vice versa with endothelial cells incorporated in 3D and cancer cells seeded in 2D on top in a fibrin gel^33^. One fully 3D system with spatially restricted cancer and vascular cells was developed in Matrigel. However, the vascular cell component was an *ex vivo* arterial ring, and angiogenic sprouting from an artery is not physiologically relevant to tumor angiogenesis^34^. These natural material systems have provided a robust platform for 2D and 3D culture of cancer and vascular cells due to the abundance of cell adhesion and degradation sites. However, natural materials have innate bioactivity that is not easy to finely tune. Conversely, the use of a PEG-based materials system allows for complete customization of the bioactivity. This material platform facilitated development of a reductionist tumor angiogenesis model with minimal bioactivity, as this work was focused on studying cell-cell interactions. Future studies can easily build on this model to explore the role of different ECM components in tumor progression because the concentrations and types of cell-adhesive ligands and degradation sites can be easily defined to match the pathophysiological tissue of interest.

In looking at mimicking processes that occur in cancer progression *in vivo*, the angiogenic switch has been defined as the point in tumor progression when cancer cells tip the balance between pro- and anti-angiogenic factor secretion, and this increase in pro-angiogenic factor secretion stimulates quiescent vasculature and leads to vascular cell migration to the tumor site^35^. Our evaluation of the release of 3 key angiogenic growth factors by 344SQ thus provided evidence that these cancer cells are pro-angiogenic in 3D in PEG hydrogels. VEGF is a well-known proangiogenic growth factor that induces endothelial cell proliferation and migration while inhibiting apoptosis^36^. Previous work has shown that tumor vascularization depends on VEGF secretion, and inhibition of VEGF disrupts tumor growth^36^,^37^. bFGF is upregulated during angiogenesis and contributes to enhancing endothelial cell migration and proliferation, as well as increasing ECM degradation^38^,^39^. Lastly, PDGF-BB facilitates pericyte recruitment to stabilize nascent vessels^40^. Thus proangiogenic growth factor secretion from 344SQ cells combined with our transwell migration assay findings suggest this paracrine signaling is likely responsible for the observed vascular cell homing to tumors within V-C hydrogels.

The increase in angiogenic growth factor secretion from TGF-β1-treated 344SQ demonstrated a correlation between 344SQ cell angiogenic capacity and metastatic progression. These findings corroborate previous studies in the literature that correlated angiogenic growth factors with severity of disease. In patients receiving treatments for metastatic cancer, elevated serum levels of bFGF and/or VEGF occurred more often in patients with progressive disease compared to patients that were responding to treatment (57% progressing patients versus 15% responding patients)^41^. In another study, high VEGF expression in primary human breast cancer tissue correlated with poor prognosis^42^. Due to the increase in size of clusters and loss of lumenization following EMT observed for 344SQ cells in PEG hydrogels, this pro-angiogenic response could be in part due to 344SQ cells experiencing hypoxia. Additionally, taken together with our findings that TGF-β1 is secreted by the vascular cell co-culture, these results suggest stimulatory feedback to the vascular cells likely occurs within our tumor angiogenesis model.

The large, disorganized 344SQ clusters observed in our model have not been previously observed for TGF-β1-treated 344SQ clusters in PEG hydrogels, whereby morphology changes have been limited to increases in size and loss of a cleared lumen^14^. In contrast, 344SQ clusters in Matrigel form invadopodia upon TGF-β1 treatment. Taken together with our finding that close proximity vascular cell interactions provide necessary cues to initiate 344SQ cluster invasion, these results demonstrate the ability of both ECM- and cell-based interactions to drive cancer cell invasion^9^. Our finding that in 3D, soluble signaling alone from vascular cells was not enough to elicit changes in cancer cluster morphology stands in contrast to one report in the literature. In this report, use of a transwell system with D492 breast epithelial cells in Matrigel in the bottom well and breast endothelial cells seeded as a monolayer in the top well was compared to the epithelial and endothelial cells cultured together in Matrigel^43^. Increases in cell clusters with mesenchymal morphology were observed when cancer cell-endothelial cell interactions were restricted to soluble signaling alone compared to direct co-culture^43^. However, this disparity could be explained by differences in model setup, whereby their vascular source was a 2D monolayer of endothelial cells and ours was a 3D co-culture of endothelial cells and pericytes. This difference in cell behavior in 2D and 3D also matches our finding that in spite of soluble signaling alone being sufficient to enhance 344SQ migration in the presence of HUVEC in a 2D transwell assay, the 3D invasive 344SQ cluster behavior required vascular cells in close proximity.

Exploration of differences in cell microenvironments in 2D and 3D can also be used to better understand mechanisms involved in the development of the large, invasive 344SQ clusters observed in our tumor angiogenesis model. *In vivo*, TGF-β1 is known to be secreted in an inactive, latent form that typically binds to the ECM and can be activated by many different factors, including proteases, low pH, and reactive oxygen species^26^,^44^. Previous work in our lab has confirmed that vascular cells co-cultured in PEG hydrogels secrete ECM components, such as collagen IV and laminin, that are deposited locally around vascular cell networks^17^. Staining for active TGF-β1 in hydrogels provided evidence that TGF-β1 was likely sequestered in vascular cell-secreted ECM and activated locally around these vascular cell networks, which could occur due to protease secretion from vascular cells during migration. Thus when vascular cells were in close proximity to cancer cells, activated TGF-β1 likely contributed to the formation of large, invasive 344SQ clusters observed at the interface. This hypothesis also accounted for why TGF-β1 secreted into the conditioned media did not lead to global changes in the morphology of 344SQ clusters throughout the hydrogel, as the clusters exhibited growth arrest, making it unlikely their quiescent, non-migratory behavior would initiate TGF-β1 activation in a concentration relevant to induce EMT^14^. Furthermore, active TGF-β1 has been previously detected around sprouting neovascular structures *in vitro* in 3D cultures and localized increases in breast cancer cell proliferation around these sprouting structures occurred, which provides additional support for our vascular cell-induced TGF-β1 activation hypothesis^45^.

Furthermore, TGF-β1 inhibition reduced 344SQ migration, a result that was consistent with a study that reported a combination of TGF-β1 and TGF-β2 neutralizing antibodies decreased endothelial cell-stimulated migration of lung carcinoma and normal mammary epithelial cells^46^. TGF-β1 inhibition reduced 344SQ migration down to a level not statistically different from the media only control, indicating TGF-β1 is a major player in vascular cell-induced 344SQ migration. This inhibitor study was conducted using a transwell assay due to compatibility issues associated with the inhibitor and the polymer system. While the transwell system is simplified, these results provided evidence in support of the hypothesis that vascular cell-secreted TGF-β1 contributed to the development of large, invasive 344SQ clusters in the tumor angiogenesis model.

Vascular cells have traditionally been studied in the context of tumor angiogenesis as the cells that facilitate tumor growth via nutrient and oxygen delivery^47^. The findings in this report indicate vascular cell interactions can elicit changes in cancer cell behavior, providing evidence that the role of vascular cells in tumor progression might not be limited to supporting growth. Very few reports investigating this hypothesis were found in the literature, where conditioned media from endothelial cells^46^ or direct co-culture and spatially restricted co-culture with endothelial cells^43^ induced morphological and migratory changes indicative of EMT in cancer cell lines. Similar studies have also shown that endothelial cells enhance growth and branching of normal breast, lung, and prostate epithelial cells in 3D culture, which provides evidence for the origin of these endothelial cell-induced changes in epithelial cells, as they may be related to signaling associated with organ development^48^,^49^,^50^. The 3D platforms used in previous studies are limited in their ability to mimic *in vivo* cell behavior because the endothelial cells in the direct co-culture platform do not spread or form networks that interact with cancer cells. However, these systems provide some relevant information to which we can compare our results. In one study, analysis was performed on MCF10A (non-cancerous breast epithelial) and MDA-MB-231 (metastatic breast cancer) cell clusters where cluster size was correlated to distance from endothelial cells. Endothelial cell proximity showed a significant effect on size of MCF10A clusters, but not MDA-MB-231 clusters^49^. While this effect was not present in the cancer cells in this study, this finding in normal breast epithelium supports our results that show vascular cell proximity-based differences in the size of lung cancer clusters. Correlations have also been made between the location of microvascular cells and increased expression of N-cadherin, a marker of EMT, in basal-like breast cancer tissue, providing evidence that our *in vitro* findings might have relevance *in vivo*^43^. One additional study we found reported that endothelial cells, both *in vitro* and *in vivo*, have the capacity to regulate breast cancer cell quiescence, whereby stable microvascular networks supported a dormant phenotype, while sprouting neovascular structures induced nearby tumor growth^45^. Overall, our results are impactful in the field of tumor angiogenesis because they provide additional evidence that vascular cells contribute to tumor progression.

While there are many advantages of our system, it is not without its limitations. We realize that while we have achieved spatially independent tumor spheroid formation and microvascular network formation, the cells can still interact via paracrine signaling, and this signaling at study initiation may not match that of tumor angiogenesis *in vivo*. However, there are many technical challenges associated with completely independent cell structure formation. Additionally, the cell choices for the model could be further improved with the use of primary cancer and vascular cells that match the tumor tissue of interest.

This research has aimed to develop a novel lung tumor angiogenesis model comprised of vascular cells and cancer cells in a fully 3D, synthetic hydrogel system with physiologically relevant spatial localization of cells. The work discussed in this report demonstrated the use of this tumor angiogenesis model to elucidate the role of vascular cells in tumor progression. The design of this model also readily facilitates further customization of matrix mechanics and biochemistry as well as incorporation of other cells important in the tumor microenvironment, thus facilitating controlled exploration of other potentially critical aspects of tumorigenesis and metastasis. These types of studies will lead to identification of novel targets for cancer therapy.

## Methods

### Cell Maintenance

HUVEC (Lonza) were cultured in EBM-2 media (Lonza) supplemented with L-glutamine (2 mM), penicillin (100 U ml^−1^), and streptomycin (100 μg ml^−1^) and the EGM-2 SingleQuot kit (Lonza): fetal bovine serum (2%), hydrocortisone, fibroblast growth factor, vascular endothelial growth factor, insulin-like growth factor, ascorbic acid, epidermal growth factor, GA-1000 (gentamicin and amphotericin-B), and heparin. HUVEC were used in studies between passages 3 and 5. HVP (Sciencell) were cultured in Pericyte Medium (Sciencell) and used between passages 3 and 10. As previously described, 344SQ cells derived from *KRas*^*G12D*^*/p53*^*R172HΔG*^ mice were cultured in RPMI 1640 media supplemented with 10% FBS (Atlanta Biologics), gentamicin (10 μg/mL), and amphotericin-B (0.25 μg/mL)^9^. The 344SQ cells were kindly provided by Dr. Jonathan M. Kurie (Professor, Department of Thoracic/Head and Neck Medical Oncology, The University of Texas M.D. Anderson Cancer Center, Houston, TX), which were derived in accordance with guidelines that were approved by the Institutional Animal Care and Use Committee at The University of Texas M.D. Anderson Cancer Center. All cells were maintained at 37 °C and 5% CO_2_.

### Material Preparation

The N-terminus of RGDS (American Peptide) was conjugated to PEG as described previously (Supplementary Fig. S1)^17^. Briefly, RGDS was mixed with monoacrylate PEG-SVA (MW 3,400 Da, Laysan Bio) in HEPBS buffer (20 mM HEPBS, 100 mM NaCl, 2 mM CaCl_2_, 2 mM MgCl_2_, pH 8.5) at a molar ratio of 1:1.2 (PEG-SVA:RGDS). The solution was maintained at pH 8 and left to react overnight at 4 °C. Following the reaction, the solution was dialyzed over the course of 2 days in a 3.5 kDa membrane mixing on a stir plate on low with 7 total water exchanges. The solution was then moved to amber vials and frozen overnight at −20 °C, followed by freezing at −80 °C for 1 hour, and then lyophilization.

A similar reaction was used to incorporate PQ into the backbone of the PEG matrix (Supplementary Fig. S1). PQ was synthesized via standard Fmoc chemistry on an APEX 396 solid phase peptide synthesizer (Aapptec) and analyzed using MALDI-ToF mass spectrometry. PQ was reacted with PEG-SVA at a molar ratio 2.1:1 (PEG-SVA:PQ) to conjugate PEG chains to both the N-terminus and the lysine residue at the C-terminus of the peptide. Dialysis and lyophilization of PEG-PQ-PEG was performed following the same procedure as done for PEG-RGDS, with the modification of using a dialysis membrane with a 6–8 kDa cutoff. Conjugation was confirmed for both peptides using gel permeation chromatography with an evaporative light scattering detector (Polymer Laboratories). For all GPC characterization experiments, in addition to running conjugated samples, PEG-SVA alone was run to identify the left-shifted conjugated peak (PEG-PQ-PEG or PEG-RGDS) and the non-shifted peak (PEG-SVA) based on overlap with the peak of PEG-SVA run alone. Conjugation efficiency was calculated as the area under the curve for the left-shifted conjugated peak over the sum of the area under the curve for the left-shifted peak and the non-shifted peak.

Fluorescently labeled PEG-RGDS was synthesized as described previously^51^. Briefly, PEG-RGDS was dissolved in sodium bicarbonate buffer (0.1 M, pH 9) and Alexa Fluor^®^ 488 carboxylic acid, 2,3,5,6-tetrafluorophenyl ester (AF488-TFP; ThermoFisher Scientific) was dissolved in dimethylsulfoxide (DMSO). AF488-TFP was added to PEG-RGDS at a 10:1 AF488-TFP:PEG-RGDS molar ratio slowly while mixing. The solution was allowed to react for 2 hr, dialyzed for 24 hr, and lyophilized.

### Cell Encapsulations in PEG-based Hydrogels

Hydrogel precursor solutions were prepared by dissolving PEG-PQ-PEG and PEG-RGDS in HEPES-buffered saline (10 mM) with 1.5% v/v triethanolamine (HBS-TEOA). The solutions were then sterilized via filtration using a 0.2 μm syringe filter and stored at −20 °C in stock concentrations. To prepare cell-laden hydrogels, PEG-PQ-PEG and PEG-RGDS precursor solutions were diluted and combined to create a polymer precursor solution of 4% w/v PEG-PQ-PEG and 3.5 mM PEG-RGDS using HBS-TEOA with the addition of 3.5 μL/mL 1-vinyl-2-pyrillidinone (Sigma) and 10 μM eosin Y photoinitiator. Cells were trypsinized, centrifuged (200× g for 5 min for HUVEC and HVP, 200× g for 4 min for 344SQ) and then resuspended in the polymer precursor solution at a concentration of 3 × 10^7^ cells/mL at a 4:1 ratio of HUVEC:HVP for V-hydrogels and 1.5 × 10^6 ^cells/mL 344SQ for C-hydrogels (Supplementary Fig. S1).

For vascular hydrogel and cancer hydrogel fabrication, cells suspended in polymer precursor solution were pipetted between 380 μm-thick polydimethylsiloxane (PDMS) spacers on a glass slide treated with Sigmacote (Sigma) to render it hydrophobic. The solution was then covered with a methacrylate-modified glass cover slip and exposed to white light for 30 s to initiate polymerization (Fiber-Lite Series 180, 150 Q halogen, Dolan Jenner). The hydrogel was subsequently removed from the Sigmacote-treated slide to yield a cell-laden hydrogel cylinder that was covalently attached to a cover slip base for easy handling. More detailed protocols on PDMS spacer fabrication, Sigmacote treatment, and glass methacrylation can be found in the Supplementary Methods.

### Immunocytochemistry

Cell-laden hydrogels were fixed in paraformaldehyde (4%) for 45 min, followed by permeabilization for 45 min in Triton X-100 (0.25%), blocking in donkey serum (5%) overnight at 4 °C, and incubation with primary antibodies diluted in PBS with 0.5% donkey serum for 40 hr at 4 °C: mouse anti-human nuclei (1:200; Millipore), rabbit anti-PECAM (1:200; Bethyl Laboratories), mouse anti-β-catenin (1:200; BD Biosciences), rabbit zonula occludens-1 (1:200; ZO-1, Invitrogen), or rabbit anti-active TGF-β1 (1:50; Promega). Following primary antibody incubation, hydrogels were rinsed with phosphate-buffered saline (PBS) containing Tween (0.01%) prior to incubation with the secondary antibodies for 40 hr at 4 °C: Alexa Fluor 488-, 555-, or 647-tagged donkey anti-mouse or -rabbit (all used at a 1:200 dilution in PBS with 0.5% donkey serum; ThermoFisher Scientific). Following antibody staining, cell-laden gels were incubated overnight in a 1:60 dilution of phalloidin (prepared according to manufacturer protocol, ThermoFisher Scientific) in PBS with 4′,6-diamidino-2-phenylindole (2 μM, DAPI) or DAPI alone. Imaging was performed on a Zeiss LSM 510 inverted confocal microscope.

### Analysis of Growth Factor Secretion from Cell-laden PEG Hydrogels

C-hydrogels were used to assess angiogenic growth factor secretion. 344SQ were cultured in hydrogels for 16 days, and for TGF-β1-treated hydrogels, incorporation of 5 ng/mL TGF-β1 (Calbiochem) was initiated on day 12 of culture. Conditioned media was collected and assessed for bFGF (Abcam), VEGF (R&D Systems), and PDGF-BB (R&D Systems) by ELISA according to the protocol for each assay kit. In addition, hydrogels were degraded at the time of media collection with collagenase (2 mg/mL, Sigma), followed by addition of RIPA buffer (Millipore) to lyse the cells. A BCA assay (ThermoFisher Scientific) was run on cell lysate to normalize growth factor concentrations to total protein in the samples. Data is shown as mean ± standard deviation. Shapiro-Wilk tests were run to confirm the normality of the data, followed by two-tailed t-tests to evaluate for statistically significant differences with an alpha level of 0.05 (n = 6 hydrogels).

V-hydrogels were used to assess TGF-β1 secretion, where they were cultured for 8 days, followed by conditioned media collection, hydrogel degradation and cell lysis as described above. Active and total TGF-β1 ELISAs were performed on the conditioned media according to the kit protocol with the use of an acid treatment to activate and detect total TGF-β1 (Promega). Active and total TGF-β1 ELISAs (Promega) were also run on conditioned media from HUVEC seeded in 2D on tissue culture polystyrene at 8 × 10^4 ^cells/cm^2^. Media samples for active TGF-β1 assessment were concentrated 6.2X with the use of Amicon Ultra 0.5 mL centrifugal filters with 10 kDa pores (Millipore).

### Transwell Migration Studies

To assess 344SQ migration in response to an angiogenic stimulus, HUVEC (8 × 10^4 ^cells/cm^2^) or EGM-2 media (for the control condition) were added to the lower chamber of a 24-well plate with hanging cell culture inserts (8 μm pore size, EMD Millipore). 24 hr later, 344SQ (1.5 × 10^5 ^cells/cm^2^) were seeded in RPMI 1640 without serum in the upper chamber and allowed to migrate for 16 hr. Media was removed, the top side of the insert was wiped with a cotton swab, and remaining migrated cells attached to the underside of the insert were fixed in 4% paraformaldehyde (Electron Microscopy Sciences) and stained with DAPI (2 μM). Migrated cells were imaged on an Axiovert 135 inverted fluorescent microscope (Zeiss) and quantified using the “Analyze Particles” macro in ImageJ software (NIH).

The same protocol was followed to assess HUVEC migration in response to cancer cells, with the following modifications. 344SQ were seeded in the lower chamber at 1.3 × 10^5 ^cells/cm^2^ and HUVEC were seeded in EGM-2 with serum in the inserts at 1 × 10^5 ^cells/cm^2^. The control condition was addition of EGM-2 to the bottom well. Data is shown as mean ± standard deviation. Shapiro-Wilk tests were run to confirm the normality of the data, followed by two-tailed t-tests to evaluate for statistically significant differences with an alpha level of 0.05 (5 images were taken per insert as repeated measures with n = 12 inserts per group for 344SQ migration and n = 6 inserts per group for HUVEC migration).

### Construction of Tumor Angiogenesis Model

To create the tumor angiogenesis model, hydrogel precursor solutions were prepared as described above. The cancer cell suspension was spiked with 7.5 μM 488-PEG-RGDS to allow for distinction between the hydrogel layers. This solution was added to a 500 μm thick PDMS well (see Supplementary Methods for protocol) on a glass slide treated with Sigmacote and exposed to white light for 20 s. A second PDMS well was stacked on top of the first and either the prepolymer solution with vascular cells (for V-C hydrogels) or acellular prepolymer (for B-C hydrogels) was added to the well and exposed to white light for 30 s. Spacers were removed and the bilayer hydrogel was cultured in EGM-2 media for 7–14 days.

### Quantitative Analysis of Changes in 344SQ Sphere Morphology

Large, disorganized 344SQ clusters appeared to occur only at the interface between vessel and cancer hydrogel layers. To confirm this observation, 4 z-stack images (635 μm thick) in different quadrants of the hydrogel were taken starting at the lowest z-position of the cancer region, as identified by the presence of 488-PEG-RGDS, for fields of view containing large, invasive structures. To ensure these changes at the interface were due to the presence of vascular cells, B-C hydrogels with an acellular layer of hydrogel in place of the vascular hydrogel were also imaged, where 4 z-stack images (635 μm thick) were taken at random positions in different quadrants of the hydrogel, starting at the lowest z-position of the cancer region.

Changes in 344SQ cluster morphology were evaluated with a macro developed using ImageJ software (NIH). The interface was identified with the use of 488-PEG-RGDS, where the average intensity in the 488-PEG-RGDS channel was plotted for each slice in the stack, and the slice with mean gray value less than 17 was identified. 10 slices above and below this slice were added to define the hydrogel interface bin. The remainder of the slices in the z-stack were divided into 105 μm-thick bins (Supplementary Fig. S6). Following binning, clusters from each hydrogel were z-projected to view the largest cross section, and binarized. A close morphological operator (structural element: circle, radius: 2 pixels) was included in the analysis to better represent cancer cluster features. Cluster area and circularity were measured for each cluster in a given bin using the “Analyze Particles” application. Clusters on the edge of the image were excluded. Also, features with area less than 400 μm^2^ were excluded to avoid measuring single cells. Manual removal of overlapping clusters, cells growing in 2D on the gel surface, and vascular cells was performed following automated analysis. Kolmogorov-Smirnov tests were run to compare the distributions for each bin to the interface bin. A Bonferroni-corrected alpha level of 0.0125 was used (3 images were taken per hydrogel as repeated measures across 5 hydrogels with 739 total clusters for V+C and 415 total clusters for B+C).

### 3D Soluble Signaling Transwell Studies

To assess the role of vascular cell soluble signaling alone on 344SQ behavior, modified PEG-based hydrogels were formed. C-hydrogels were polymerized on methacrylate-modified glass and added to a well plate. V-hydrogels were polymerized without attachment to glass and placed in transwell inserts (EMD Millipore) hanging above the cancer hydrogels (Fig. 7a). A pore size of 0.4 μm was chosen to enable the diffusion of soluble factors while preventing cell migration. Staining with DAPI and phalloidin was performed as described above and imaging was performed on a Zeiss LSM 510 inverted confocal microscope, whereby z-stack images were taken through the thickness of each hydrogel. Hydrogels were divided into 105 μm-thick bins according to distance from the top of the hydrogel to account for possible differences in cell behavior for 344SQ clusters in closest proximity to the vascular cell stimulus. Analysis was performed similar to what was described above for V-C and B-C hydrogels, whereby clusters were z-projected, binarized, and circularity and area for each cluster was measured using the “Analyze Particles” application. Kolmogorov-Smirnov tests were run to compare the distributions for each bin to the interface bin. A Bonferroni-corrected alpha level of 0.0167 was used (4 images were taken per hydrogel as repeated measures; n = 4 hydrogels per group).

### TGF-β1 Inhibitor Transwell Migration Assay

To investigate whether TGF-β1 inhibition could prevent vascular cell-induced 344SQ migration, the 2D transwell assay described above was performed with a few modifications. In addition to the HUVEC and EGM-2 media groups, an additional HUVEC group was included and 24 hr after seeding, media was changed to incorporate the TGF-β1 inhibitor, SB431542 (Sigma), into one of the HUVEC groups. SB431542 in DMSO was added to EGM-2 at 25 μM and 1 mL was added to each well of the HUVEC-seeded treatment group. As a control, the same volume of DMSO was added to EGM-2 media and 1 mL was added to each well of the HUVEC-seeded control group. Migration time and analysis approach were the same as described above. Data is shown as mean ± standard deviation. Shapiro-Wilk tests were run to confirm the normality of the data, followed by an analysis of variance (ANOVA), and pairwise comparisons with a Tukey-Kramer HSD test using an alpha level of 0.05 (5 images were taken per insert as repeated measures; n = 5 inserts per group).

## Additional Information

**How to cite this article**: Roudsari, L. C. *et al*. A 3D Poly(ethylene glycol)-based Tumor Angiogenesis Model to Study the Influence of Vascular Cells on Lung Tumor Cell Behavior. *Sci. Rep.* **6**, 32726; doi: 10.1038/srep32726 (2016).

## Supplementary Material

Supplementary Information
