# Supplementary material for: A 3D Poly(ethylene glycol)-based Tumor Angiogenesis Model to Study the Influence of Vascular Cells on Lung Tumor Cell Behavior
Source: Sci Rep. 2016 Sep 6;6:32726. doi: 10.1038/srep32726 (PMC5011743; doi:10.1038/srep32726)
Supplement: Supplementary Information [file srep32726-s1.pdf]

A 3D Poly(ethylene glycol)-based Tumor Angiogenesis Model to Study the Influence of Vascular Cells on Lung Tumor Cell Behavior

Laila C. Roudsari[1], Sydney E. Jeffs[1], Amber S. Witt[1], Bartley J. Gill[2], Jennifer L. West*[1]

[1]Department of Biomedical Engineering, Duke University, Durham, North Carolina,

[2]Department of Bioengineering, Rice University, Houston, Texas

SUPPLEMENTARY INFORMATION

SUPPLEMENTARY METHODS

Sigmacote Treatment of Glass Slides

In order to create a hydrophobic surface to repel PEG during polymerization, glass slides were treated with Sigmacote (Sigma). In brief, glass slides were washed with DI water and rinsed with acetone to clean. After allowing slides to dry, they were then submerged in Sigmacote, which was followed immediately by rinsing with 70% ethanol, and wiping to dry. Submersion, rinsing, and drying was repeated 5 times for each slide.

Modification of Glass with Methacrylate Groups

Cover slips were cleaned in Piranha solution (H_2SO_4 and H_2O_2) for 1 hr, followed by rinsing with water and then ethanol. Methacrylation was then performed via treatment with 2% 3-(trimethoxysilyl)propyl methacrylate in ethanol for 3 days. Coverglass were then washed with ethanol, and baked for 1 hr at 60℃.

Production of PDMS Spacers and Wells

Elastomer base and elastomer curing agent (Dow Corning) were mixed together in a 10:1 ratio (base:curing agent). The solution was degassed to remove bubbles and then pipetted between 2 Sigmacote-treated glass slides with a Teflon spacer to create a polydimethylsiloxane (PDMS) slab of desired thickness. The solution was then baked overnight at 60℃. Following curing, the slab was removed from the glass slides. Spacers were created by cutting strips from the slab and wells were created using a circular metal punch with 3.8 mm inner diameter.

Cytotoxicity Assessment of 344SQ Cells

In order to ensure the 50 s polymerization time of the cancer hydrogel layer in the tumor angiogenesis model did not negatively affect 344SQ cell viability, B-C hydrogels were fabricated. After 48 hr in culture, media was removed and cell-laden hydrogels were incubated in Live/Dead staining solution (ThermoFisher Scientific) prepared according to manufacturer's instructions for 50 min. Immediately following staining, imaging was performed on a Zeiss LSM 510 inverted confocal microscope.

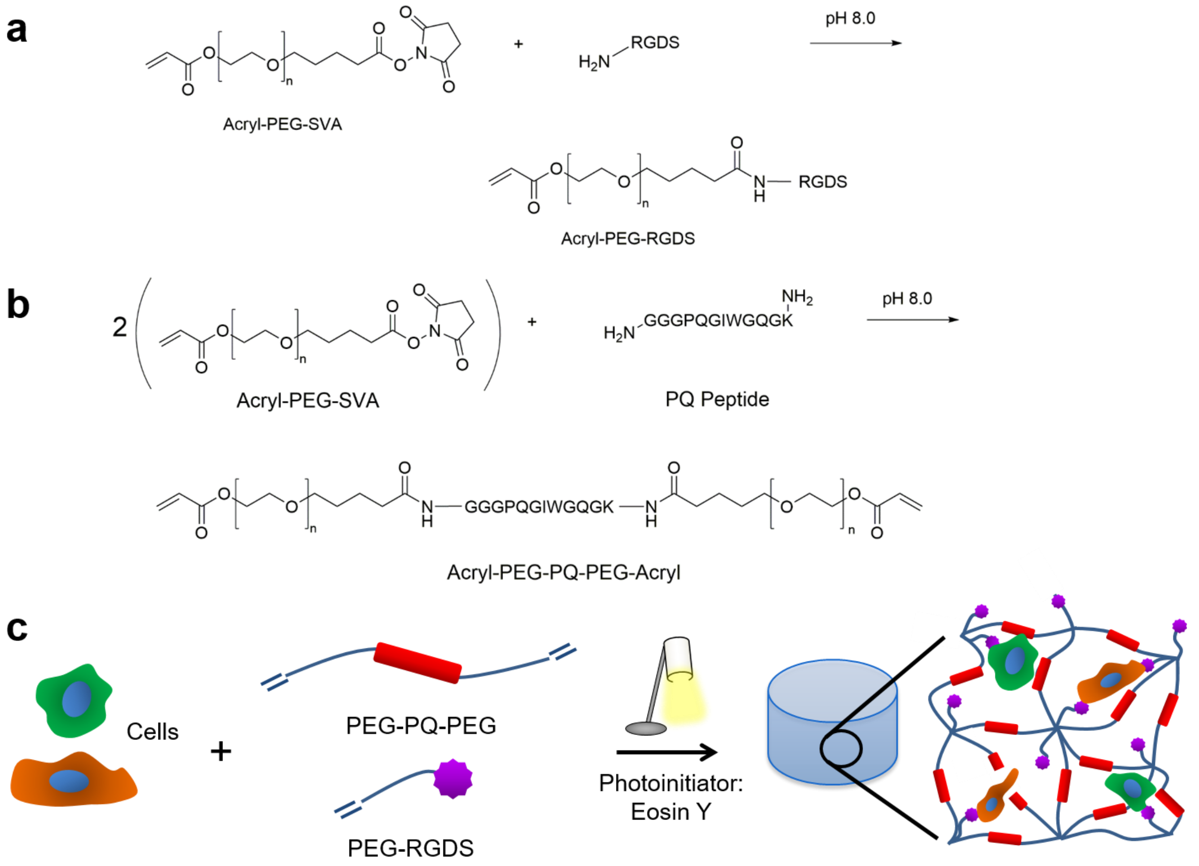

Figure S1: PEG-peptide conjugations and schematic of cell encapsulation in PEG hydrogels. Reaction scheme for PEG conjugation to (a) RGDS and (b) GGGPQGIWGQGK (PQ). (c) Cells are mixed with prepolymer solution (PEG-PQ-PEG and PEG-RGDS) and eosin Y photoinitiator and exposed to white light for photopolymerization, resulting in the formation of cell-adhesive, proteolytically degradable hydrogels with cells encapsulated in 3D.

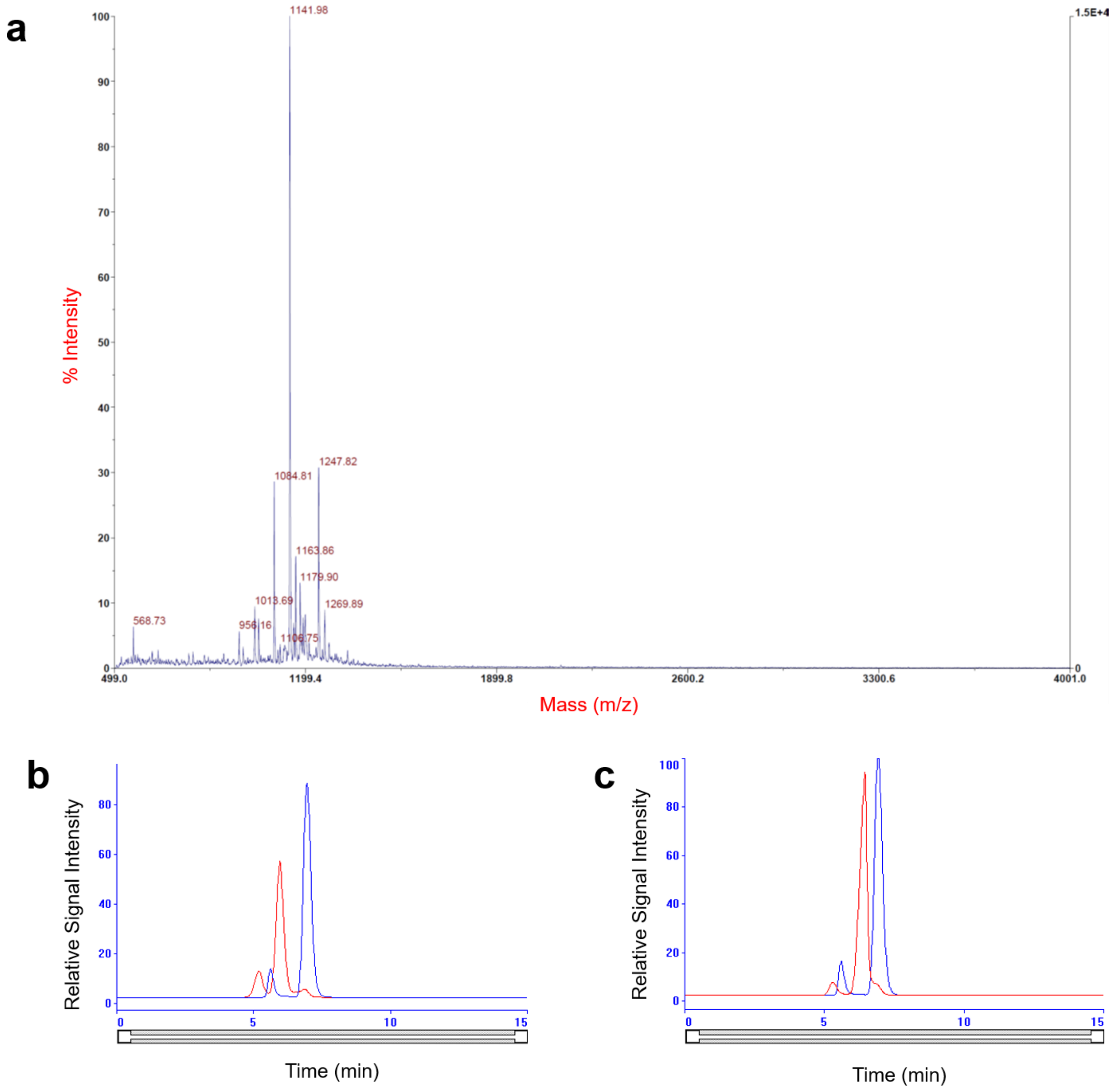

Figure S2: Materials characterization. (a) MALDI-ToF mass spectrometry trace of GGGPQGIWGQGK (PQ) peptide. Representative gel permeation chromatography traces for (b) PEG-PQ-PEG and (c) PEG-RGDS, from which conjugation efficiencies were calculated (red – PEG-peptide; blue – PEG-SVA).

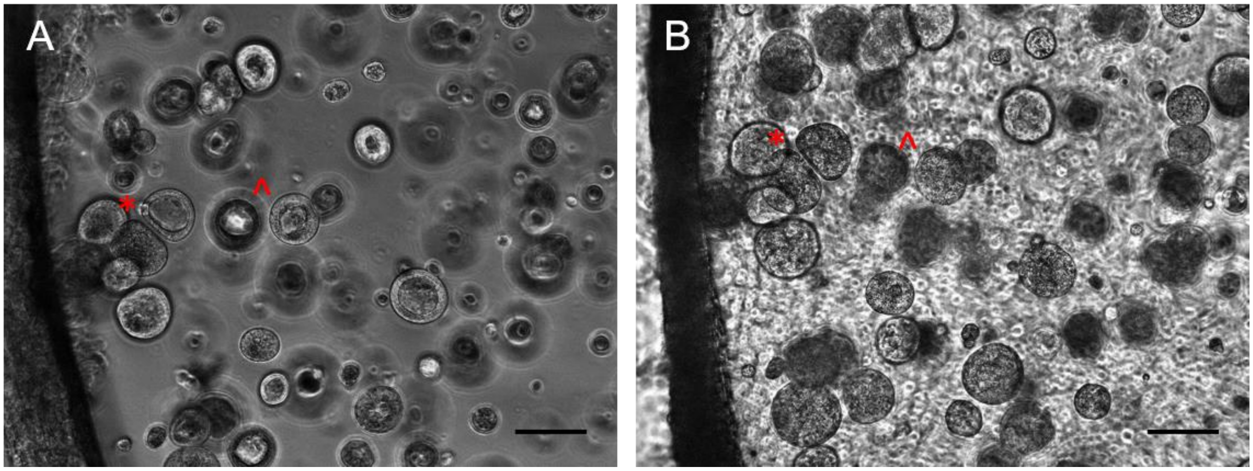

Figure S3: 344SQ cell response to TGF-β1 exposure in PEG hydrogels. (a) An image of 344SQ cells at day 12 in culture. (b) The same region of the hydrogel was imaged at day 16 in culture, following 4 days of treatment with 5 ng/mL TGF- β1 (red * and red ^ indicate the same spheroids of the hydrogel; scale bars = 100 μm).

a

b

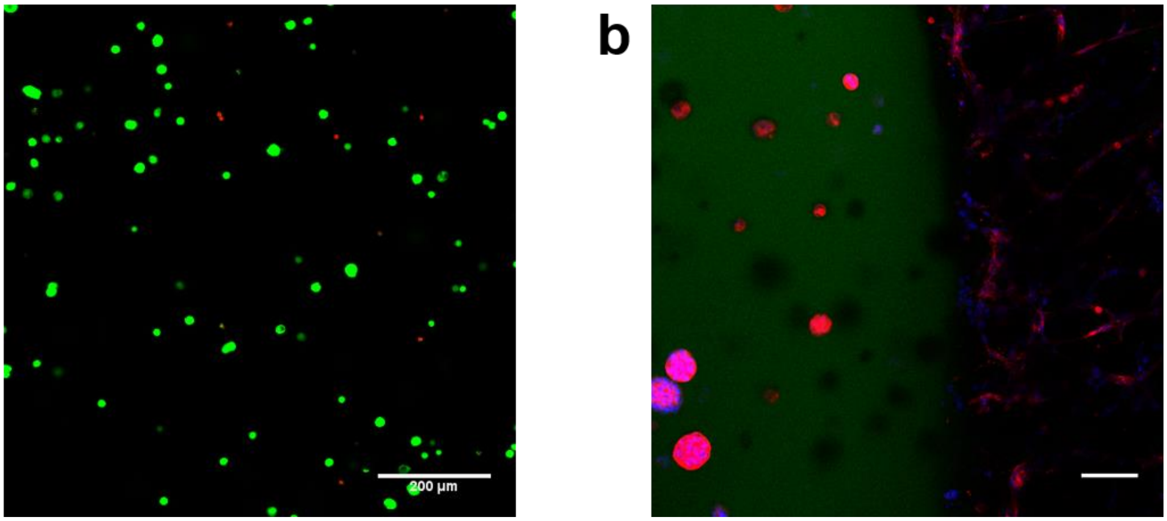

Figure S4: Tumor angiogenesis model validation. (a) Live/dead staining of cancer only control model after 48 hr in culture to show high viability of cells after 50 s of light exposure (green – calcein AM, red – ethidium homodimer; scale bar = 200 μm). (b) Image of tumor angiogenesis model at day 3, viewing the hydrogel orthogonal to the interface, depicting the presence of 344SQ clusters in the Alexafluor 488-PEG-RGDS-positive region of the hydrogel and vascular network formation in the non Alexafluor 488-PEG-RGDS-positive region of the hydrogel (red – phalloidin, blue – DAPI, green – AF488-PEG-RGDS; scale bar = 100 μm).

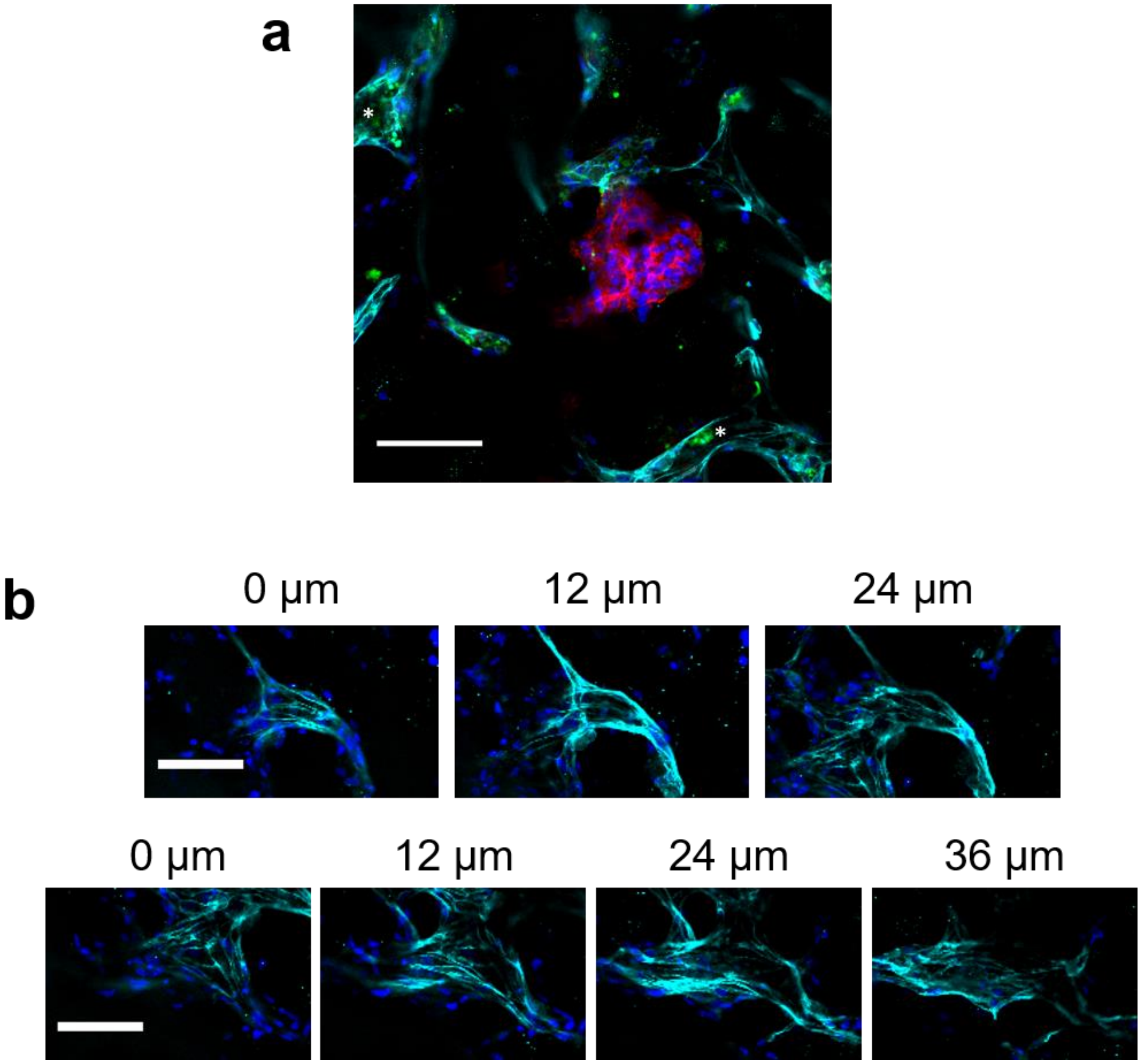

Figure S5: Confirmation of lumens in vascular networks within V-C hydrogels. (a) An image confirming lumenal clearing has occurred in vascular structures in V-C hydrogels (lumens indicated with *; red – phalloidin, blue – DAPI, cyan– PECAM, green – human nuclei; scale bar = 100 µm). (b) Slices from z-stacks are shown for vascular structures in V-C hydrogels confirming lumens in V-C hydrogels (cyan – PECAM, blue – DAPI; scale bar = 100 µm).

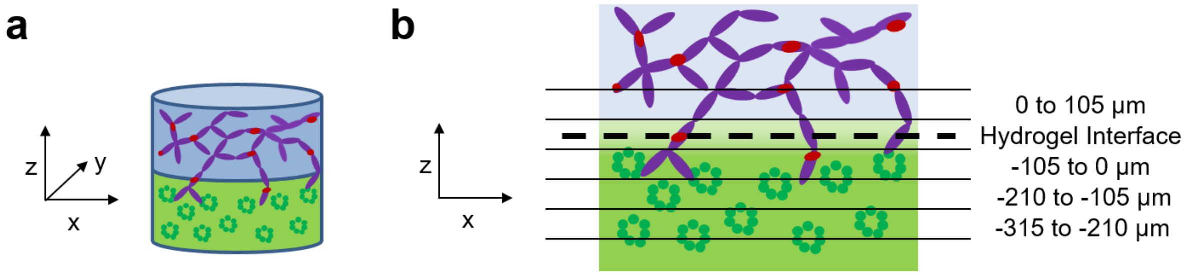

Figure S6: Tumor angiogenesis model schematics depicting binning for quantification. (a) Schematic of tumor angiogenesis model with 488-PEG-RGDS. (b) Hydrogel binning for quantification: z-stack images were taken through the hydrogel. 488-PEG-RGDS was used to identify the hydrogel interface slice by plotting the average intensity for each slice and using a defined intensity threshold for the interface. 10 slices above and below the interface slice were included to define the interface bin and remaining slices were divided into 105 μm-thick bins.
